# Cell-to-Cell Spreading of HIV-1 in Myeloid Target Cells Escapes SAMHD1 Restriction

**DOI:** 10.1128/mBio.02457-19

**Published:** 2019-11-19

**Authors:** Maorong Xie, Héloïse Leroy, Rémi Mascarau, Marie Woottum, Maeva Dupont, Camille Ciccone, Alain Schmitt, Brigitte Raynaud-Messina, Christel Vérollet, Jérôme Bouchet, Lucie Bracq, Serge Benichou

**Affiliations:** a Inserm U1016, Institut Cochin, Paris, France; b CNRS, UMR8104, Paris, France; c Université Paris-Descartes, Sorbonne Paris-Cité, Paris, France; d International Associated Laboratory (LIA VirHost), CNRS, Université Paris-Descartes, Inserm, and Institut Pasteur Shanghai—Chinese Academy of Sciences, Shanghai, China; e Institut de Pharmacologie et de Biologie Structurale, CNRS, Université Paul Sabatier, Toulouse, France; f Institut Pasteur Shanghai—Chinese Academy of Sciences, Shanghai, China; University of California, San Diego; University of Pittsburgh School of Medicine

**Keywords:** HIV-1, SAMHD1, cell fusion, myeloid cells, virus spreading

## Abstract

Dendritic cells (DCs) and macrophages as well as osteoclasts (OCs) are emerging as target cells of HIV-1 involved in virus transmission, dissemination, and establishment of persistent tissue virus reservoirs. While these myeloid cells are poorly infected by cell-free viruses because of the high expression levels of cellular restriction factors such as SAMHD1, we show here that HIV-1 uses a specific and common cell-to-cell fusion mechanism for virus transfer and dissemination from infected T lymphocytes to the target cells of the myeloid lineage, including immature DCs (iDCs), OCs, and macrophages, but not monocytes and mature DCs. The establishment of contacts with infected T cells leads to heterotypic cell fusion for the fast and massive transfer of viral material into OC and iDC targets, which subsequently triggers homotypic fusion with noninfected neighboring OCs and iDCs for virus dissemination. These two cell-to-cell fusion processes are not restricted by SAMHD1 and allow very efficient spreading of virus in myeloid cells, resulting in the formation of highly virus-productive multinucleated giant cells. These results reveal the cellular mechanism for SAMHD1-independent cell-to-cell spreading of HIV-1 in myeloid cell targets through the formation of the infected multinucleated giant cells observed *in vivo* in lymphoid and nonlymphoid tissues of HIV-1-infected patients.

## Abstract (precis)

We demonstrate that HIV-1 uses a common two-step cell-to-cell fusion mechanism for massive virus transfer from infected T lymphocytes and dissemination to myeloid target cells, including dendritic cells and macrophages as well as osteoclasts. This cell-to-cell infection process bypasses the restriction imposed by the SAMHD1 host cell restriction factor for HIV-1 replication, leading to the formation of highly virus-productive multinucleated giant cells as observed *in vivo* in lymphoid and nonlymphoid tissues of HIV-1-infected patients. Since myeloid cells are emerging as important target cells of HIV-1, these results contribute to a better understanding of the role of these myeloid cells in pathogenesis, including cell-associated virus sexual transmission, cell-to-cell virus spreading, and establishment of long-lived viral tissue reservoirs.

## INTRODUCTION

Myeloid cells, including subsets of blood monocytes, dendritic cells (DCs), macrophages, and bone osteoclasts (OCs), express the CD4 receptor and CCR5 and CXCR4 coreceptors required for HIV-1 entry and represent natural host cell targets of the virus (1, 2). However, most of the attention regarding HIV-1 spreading and persistence has been focused on T lymphocytes, even if myeloid cells are now emerging as important target cells involved in all steps of HIV-1 pathogenesis and in viral persistence in tissues of infected individuals, even under conditions of antiretroviral treatment (3). Human myeloid lineage cells play pivotal roles during infection as demonstrated in HIV-infected patients, simian immunodeficiency virus (SIV)-infected macaques, and, more recently, in HIV-1-infected humanized mice (3–12). While DCs have been proposed to be the first cells targeted by HIV-1 after sexual transmission and are involved in early virus dissemination in lymphoid organs, where they can transmit viral particles to activated CD4^+^ T cells (13–16), macrophages participate in the formation of virus reservoirs in numerous host lymphoid and nonlymphoid tissues (9, 10, 12, 17–20). Finally, infection of OCs, which are cells specialized in bone resorption, has been studied recently, and this is likely responsible for the bone disorders observed in HIV-1-infected patients (21, 22).

While HIV-1 infection with cell-free viruses has been largely documented, cell-to-cell transmission probably represents the dominant mode of infection (23, 24). This propagation route is very efficient and involves the different cell types targeted by HIV-1, including cells of the myeloid lineage (i.e., macrophages, DCs, and OCs) (22, 24–26), which are not easily infected by cell-free viruses, mainly because of the high expression of cellular restriction factors, including SAMHD1, an enzyme that cleaves deoxynucleoside triphosphates (dNTPs) and depletes the pool of intracellular nucleotides necessary for efficient HIV-1 replication in these noncycling myeloid cells (16, 27–31). This cell-to-cell mode of infection likely plays important roles at the level of genital and rectal mucosa and then for virus spread in numerous host tissues. However, there is still a paucity of knowledge of the mechanisms that control virus dissemination by cell-to-cell transfer in myeloid cell targets. Only two recent reports have been published regarding virus cell-to-cell dissemination to macrophages from infected T cells. While it was reported previously by the group of Quentin Sattentau that macrophages could be infected via selective capture or phagocytosis of HIV-1-infected T cells (26), we have reported that HIV-1 is mainly transferred to macrophages from infected T cells by a two-step cell fusion process (25).

Since myeloid cells are emerging as important target cells of HIV-1, the goal of this study was to understand the mechanisms involved in cell-to-cell transfer of HIV-1 from infected T cells for virus spreading in myeloid cells. We show that HIV-1 uses a two-step cell fusion process for virus transfer from infected T lymphocytes to myeloid cells, including macrophages, OCs, and immature DCs (iDCs), and then subsequent dissemination in these target cells, leading to the formation of highly virus-productive multinucleated giant cells (MGCs). These results reveal a very efficient mechanism for virus spreading in myeloid cell targets and the formation of MGCs *in vivo* in tissues of HIV-1-infected patients.

## RESULTS

### Efficient HIV-1 cell-to-cell transfer between infected T cells and myeloid cells.

**FIG 1:**
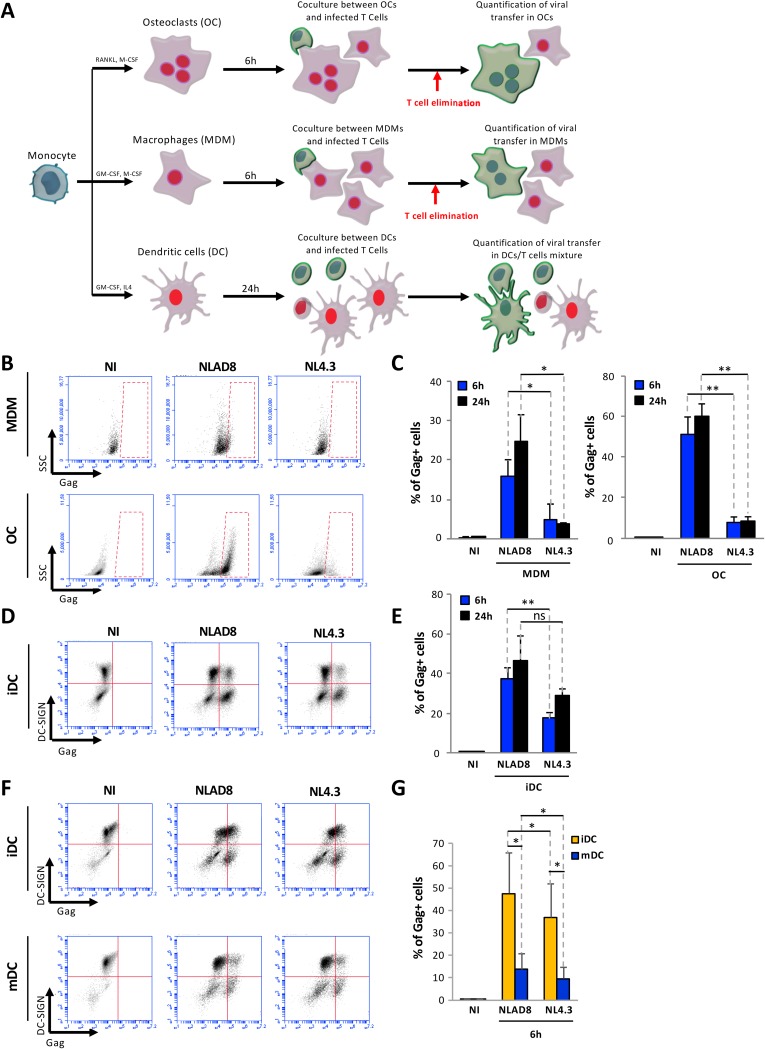
HIV-1 cell-to-cell transfer from infected T cells to myeloid cells. (A) Experimental protocol. (B) Jurkat cells were infected with the NLAD8 or NL4.3 strains for 36 h and then cocultured for 6 or 24 h with MDMs or OCs. (C) After elimination of Jurkat cells, the percentage of Gag^+^ MDMs or OCs was determined by flow cytometry. As a negative control (NI), noninfected Jurkat cells were cocultured with MDMs or OCs. (D) Jurkat cells were infected as described above and then cocultured with iDCs for 6 or 24 h. (E) The percentage of Gag^+^/DC-SIGN^+^ iDCs was evaluated by flow cytometry. (F) Jurkat cells were infected and then cocultured with iDCs or mDCs for 6 h. (G) The percentage of Gag^+^/DC-SIGN^+^ iDCs or mDCs was evaluated by flow cytometry. The results represent means of at least 4 independent experiments performed with MDMs, OCs, iDCs, or mDCs of 4 different donors. Error bars represent 1 standard error of the mean (SEM). Statistical significance was determined using the Mann-Whitney U-test (ns, *P* > 0.05; *, *P* < 0.05; **, *P* < 0.01).

Since we and others have previously reported that HIV-1 could be efficiently transferred from T cells to macrophages through cell-to-cell contacts, we investigated whether HIV-1 could also be transferred to other myeloid cell targets, i.e., DCs and OCs. CD14^+^ monocytes were isolated from blood donors; differentiated in macrophages (monocyte-derived macrophages [MDMs]), OCs, or immature DCs using specific cytokine cocktails; and used as target cells for coculture with infected CD4^+^ T cells (i.e., Jurkat CD4^+^ T cells or autologous purified human primary CD4^+^ T cells) as schematized in Fig. 1A. Differentiated cells were characterized morphologically and functionally and by differential expression of specific markers (see Fig. S1 in the supplemental material). To analyze virus cell-to-cell transfer between infected T cells and cells of the myeloid lineage, Jurkat cells or primary T cells were infected with CCR5-using macrophage-tropic (NLAD8) virus or CXCR4-using lymphotropic (NL4.3) virus and then cocultured with MDMs, OCs, or iDCs for 6 or 24 h (Fig. 1). Since MDMs and OCs were strongly adherent, T cells were eliminated by extensive washes, and MDMs and OCs were collected and stained for the intracellular viral Gag antigen. The percentage of Gag-positive (Gag^+^) cells was then determined by flow cytometry (Fig. 1B and C). As expected, around 15% of the MDMs exhibited high levels of Gag expression after 6 h of coculture with NLAD8-infected T cells. Interestingly, around 50% of the OCs were already Gag^+^ as soon as 6 h of coculture with NLAD8-infected T cells, indicating very efficient viral transfer from infected T cells to OCs. In comparison, a very low (less than 5%) level of viral transfer was detected in MDMs or OCs cocultured with NL4.3-infected T cells. Regarding viral transfer to iDCs, which are semiadherent cells, iDCs and T cells were collected after coculture (see Fig. 1A) and stained for intracellular Gag and cell surface dendritic cell-specific intercellular adhesion molecule-3-grabbing nonintegrin (DC-SIGN). The percentage of Gag^+^ cells was then analyzed in the DC-SIGN^+^ cell population (Fig. 1D and E). Significant (20% to 50%) levels of Gag^+^/DC-SIGN^+^ cells were detected by flow cytometry when iDCs were cocultured with either NLAD8- or NL4.3-infected T cells, even if the levels of Gag^+^/DC-SIGN^+^ cells were significantly higher in iDCs cocultured with T cells infected with the NLAD8 macrophage-tropic virus than with NL4.3-infected T cells. Finally, we also compared levels of virus transfer to monocyte-derived DCs from the same donors before cell maturation (iDCs) or after maturation (mDCs) induced by bacterial lipopolysaccharide (LPS) treatment (Fig. S1E). iDCs and mDCs were thus cocultured for 6 h with infected T cells, and viral transfer was analyzed as before after Gag and DC-SIGN staining. While both viruses were efficiently transferred to iDCs, virus transfer was significantly reduced in mDCs from the same donors (Fig. 1F and G), showing that iDCs are more susceptible to HIV-1 cell-to-cell transfer from infected T cells.

10.1128/mBio.02457-19.1 FIG S1 Characterization of cells of the myeloid lineage. (A to C) CD14^+^ blood monocytes were differentiated in MDMs or in OCs, and the levels of expression of the indicated OC-specific factors were analyzed by RT-qPCR (A). The results represent means of 10 independent experiments performed with MDMs or OCs from 10 donors. (B) MDMs and OCs were stained with phalloidin (green) and DAPI (blue) and analyzed by confocal microscopy (scale bars, 5 μm), and fusion index values (right panel) was determined by quantification of at least 500 cells from 5 different donors. (C) MDMs or OCs were seeded on bovine bone slides and stained with toluidine blue. Bone resorption pits were analyzed using bright-field images (scale bars, 20 μm), and bone degradation index values (right panel) were determined for 7 different donors. (D and E) Monocytes were differentiated in MDMs (gray bars) (D) or iDCs (red bars) (E). Maturation of DCs (mDCs; green bars) was induced using LPS. Expression of the indicated MDM and DC markers was analyzed by flow cytometry. The results shown are representative of 4 independent experiments performed with DCs and MDMs from 4 different donors. Error bars represent 1 SEM. Statistical significance was determined using the Mann-Whitney U-test (ns, *P* > 0.05; *, *P* < 0.05; **, *P* < 0.01; ***, *P* < 0.001). Download FIG S1, PDF file, 0.1 MB. Copyright © 2019 Xie et al. 2019 Xie et al. This content is distributed under the terms of the Creative Commons Attribution 4.0 International license.

Together, the results presented in Fig. 1 show that, in addition to macrophages, other myeloid cell targets, including OCs and iDCs, can support significant transfer of viral material from infected T cells.

### Cell-to-cell transfer of macrophage-tropic virus by heterotypic cell fusion between T cells and myeloid cells.

**FIG 2:**
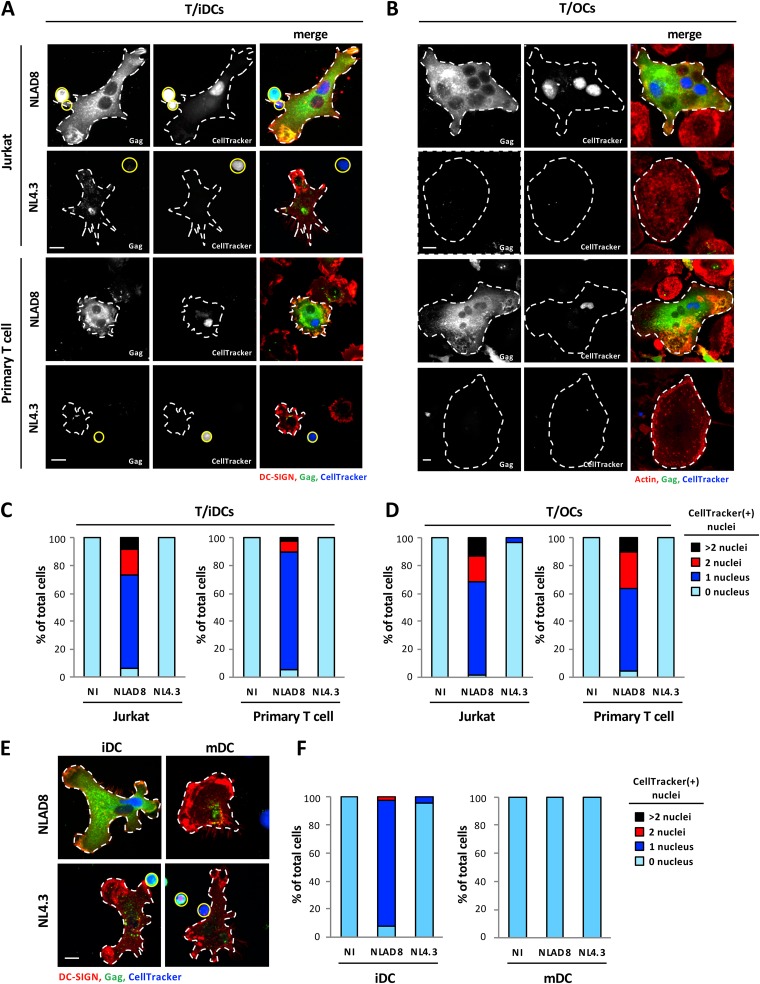
Viral transfer to immature DCs and OCs by cell fusion with infected T cells. (A and C) NLAD8- or NL4.3-infected Jurkat (A, upper images; C, left panel) or primary CD4^+^ T (A, lower images; C, right panel) cells prelabeled with CellTracker were cocultured for 6 h with iDCs. Cells were then stained with anti-Gag and anti-DC-SIGN and analyzed by confocal microscopy (scale bar, 10 μm) (A). T cells and iDCs are indicated by plain yellow and dashed white lines, respectively. (C) The number of nuclei per DC-SIGN^+^ cell was determined from images of at least 50 cells. Results are expressed as the percentages of cells with 0, 1, 2, or more than 2 CellTracker-positive [CellTracker(+)] nuclei. (B and D) NLAD8- or NL4.3-infected Jurkat (B, upper images; D, left panel) or primary CD4^+^ T (B, lower images; D, right panel) cells prelabeled with CellTracker were cocultured with OCs for 6 h. After elimination of T cells, OCs were stained with anti-Gag and phalloidin (actin) and analyzed by confocal microscopy (scale bar, 10 μm) (B). OCs are indicated by dashed white lines. (D) The number of nuclei per cell was determined from images of at least 50 cells. Results are expressed as the percentages of cells with 0, 1, 2, or more than 2 CellTracker(+) nuclei. (E and F) NLAD8- or NL4.3-infected Jurkat cells prelabeled with CellTracker were cocultured for 6 h with iDCs (E, left images; F, left panel) or mDCs (E, right images; F, right panel). Cells were then stained with anti-Gag and anti-DC-SIGN and analyzed by confocal microscopy (scale bar,10 μm) (E). T cells and DCs are indicated by plain yellow and dashed white lines, respectively. (F) The number of nuclei per DC-SIGN^+^ cell was determined from images of at least 50 cells. Results are expressed as the percentages of cells with 0, 1, 2, or more than 2 CellTracker(+) nuclei. The results shown are representative of 3 independent experiments performed with DCs and OCs from 3 different donors. NI, noninfected Jurkat or primary CD4^+^ T cells were cocultured with OCs or DCs.

Since we previously showed that HIV-1 is mainly transferred from infected T cells to macrophages through heterotypic cell fusion (25), we then performed immunofluorescence analysis to visualize virus transfer from infected T cells to DCs and OCs. Infected Jurkat or primary T cells were preloaded with the CellTracker dye for staining of T cell nuclei and were cocultured with DCs or OCs for 6 h. Cells were then stained for intracellular Gag and by the use of DRAQ5 dye to stain all nuclei (not shown) before observation by fluorescence microscopy (Fig. 2; see also Fig. S2). After 6 h of coculture with NLAD8-infected T cells, iDCs and OCs exhibited strong and diffuse cytoplasmic Gag staining (Fig. 2A and B). Interestingly, more than 95% of Gag^+^ iDCs contained several nuclei, with the average numbers of nuclei being 3.3 (Jurkat/iDCs) and 2.9 (primary T cells/iDCs), when they were cocultured with NLAD8-infected T cells (Fig. S2A, left and middle panels). Similar results were obtained with iDCs cocultured with autologous primary T cells infected with other macrophage-tropic viral strains such as strains YU2 and JRFL (Fig. S3). As shown in Fig. 2B, diffuse Gag staining was also observed when OCs were cocultured with NLAD8-infected T cells and were found to contain several nuclei. Almost all these Gag^+^ multinucleated iDCs and OCs contained at least 1 CellTracker dye-stained nucleus coming from T cells (Fig. 2A to D; see also Fig. S3B) and a total number of nuclei significantly higher than that seen with uninfected cells (Fig. S2A to C), demonstrating cell fusion between infected T cells and iDCs or OCs resulting in the formation of Gag^+^ MGCs. In contrast, when iDCs and OCs were cocultured with the lymphotropic NL4.3-infected T cells, no cell fusion was observed and they did not contain CellTracker-positive nuclei (Fig. 2A to D; see also Fig. S2A and B). Interestingly, mononucleated Gag^+^ iDCs cocultured with NL4.3-infected T cells showed cytoplasmic dotted Gag staining (Fig. 2A; see also Fig. S4A), suggesting that lymphotropic viruses can be captured by iDCs and accumulate in cytoplasmic membrane compartments after cell-to-cell contact, as previously shown for cell-free viruses (32, 33). Of note, dotted Gag staining was also observed in a few mononucleated iDCs cocultured with NLAD8-infected T cells (Fig. S4A). In agreement with this observation, we were able to visualize accumulation of mature virus particles in virus-containing compartments and plasma membrane invaginations by transmission electron microscopy when iDCs were cocultured with NLAD8- or NL4.3-infected T cells (Fig. S4F). Similarly, when LPS-induced mDCs were cocultured with NLAD8- or NL4.3-infected T cells, no cell fusion with T cells was observed, and mDCs contained only a single CellTracker-negative [CellTracker(-)] nucleus (Fig. 2E and F; see also Fig. S2C). However, virus capture by mDCs, indicated by cytoplasmic dotted Gag staining, was observed after coculture with NLAD8- or NL4.3-infected T cells (Fig. 2E). Finally, we never observed phagocytosis events of infected T cells by iDCs, mDCs or OCs such as were reported previously for virus cell-to-cell transfer to macrophages from dying infected T cells (26).

10.1128/mBio.02457-19.2 FIG S2 Formation of multinucleated cells through cell fusion between infected T cells and iDC or OC cell targets. (A and B) NLAD8- or NL4.3-infected Jurkat or primary CD4^+^ T cells prelabeled with CellTracker were cocultured for 6 h with iDCs (A) or OCs (B). Cells were then stained with anti-Gag, in combination with anti-DC-SIGN or phalloidin (actin), and analyzed by confocal microscopy. The number of nuclei per iDC and OC cell was determined from images of at least 50 cells. Results are expressed as the percentage of cells with 1, 2, 3, or more than 3 nuclei (left panels), as the mean number of nuclei per cell (central panels), and as the percentage of cells with 1, 2, 3, or more than 3 CellTracker-negative [CellTracker(-)] nuclei (right panels). (C) NLAD8- or NL4.3-infected Jurkat cells prelabeled with CellTracker were cocultured for 6 h with iDCs or mDCs. Cells were then stained with anti-Gag and anti-DC-SIGN and analyzed by confocal microscopy. The number of nuclei per DC-SIGN^+^ cell was determined from images of at least 50 cells. Results are expressed as the percentages of iDCs and mDCs with 1, 2, 3, or more than 3 nuclei (left and central panels, respectively) and as the mean number of nuclei per iDC or mDC (right panel). The results shown are representative of at least 3 independent experiments performed with DCs and OCs from at least 3 different donors. NI, noninfected Jurkat or primary CD4^+^ T cells were cocultured with OCs or DCs. Statistical significance was determined using the Mann-Whitney U-test (ns, *P* > 0.05; ****, *P* < 0.0001). Download FIG S2, PDF file, 1.6 MB. Copyright © 2019 Xie et al. 2019 Xie et al. This content is distributed under the terms of the Creative Commons Attribution 4.0 International license.

10.1128/mBio.02457-19.3 FIG S3 Transfer of macrophage-tropic viruses to iDCs by cell fusion. Primary CD4^+^ T cells infected with viral strain NLAD8, JRFL, or YU2 were prelabeled with CellTracker and cocultured for 6 h with iDCs. Cells were then stained with anti-Gag, anti-DC-SIGN, and DRAQ5 and analyzed by confocal microscopy (scale bar, 20 μm). Representative images are shown in panel A. The number of nuclei per DC-SIGN^+^ cell was determined from images of at least 50 cells. (B) Results are expressed as the percentages of cells with 0, 1, 2, or more than 2 CellTracker(+) nuclei. (C) Results are expressed as the percentage of cells with 1, 2, 3, or more than 3 nuclei (left panel), as the mean number of nuclei per cell (central panel), and as the percentage of cells with 1, 2, 3, or more than 3 CellTracker(-) nuclei (right panel). Download FIG S3, PDF file, 0.4 MB. Copyright © 2019 Xie et al. 2019 Xie et al. This content is distributed under the terms of the Creative Commons Attribution 4.0 International license.

10.1128/mBio.02457-19.4 FIG S4 Characterization of virus capture and transfer without cell fusion by DC, monocyte, and CD4^+^ T cell targets. (A) NLAD8- or NL4.3-infected Jurkat cells prelabeled with CellTracker were cocultured for 6 h with iDCs and then stained with anti-Gag, phalloidin (actin), and DRAQ5 (Nuclei). Cells were analyzed by confocal microscopy (scale bar, 10 μm). (B and C) NLAD8- or NL4.3-infected Jurkat cells prelabeled with CellTrace were cocultured for 6 h with primary monocytes and then stained with anti-Gag, phalloidin (actin), and DRAQ5 (Nuclei) and analyzed by confocal microscopy (B; scale bar, 10 μm). T cells and monocytes are indicated by plain yellow and dashed white lines, respectively. (D and E) NLAD8- or NL4.3-infected primary CD4^+^ T cells preloaded with CellTracker were cocultured for 6 h with primary CD4^+^ T cells and then stained with anti-Gag, phalloidin (actin), and DRAQ5 (Nuclei) and analyzed by confocal microscopy (D; scale bars, 10 μm). (C and E) The number of nuclei per monocyte (C) or T cell (E) target was determined from images of at least 100 cells, and the results are expressed as the percentage of cells containing 1 or 2 nuclei. The results shown are representative of 3 independent experiments performed with monocytes or CD4^+^ T cell targets from 3 different donors. NI, noninfected Jurkat or primary T cells were cocultured with monocytes or primary T cell targets. (F) NLAD8- or NL4.3-infected Jurkat cells were cocultured for 6 h with iDCs, and cells were then fixed and dehydrated. Ultrathin sections were cut, stained, and observed with a transmission electron microscope. The images at the right (scale bar, 200 nm) correspond to higher magnification of the areas indicated by the red squares in the images at the left (scale bars, 0.5 μm). The results shown are representative of 3 independent experiments performed with iDCs from 3 donors. Virus particles are indicated by red arrows. Download FIG S4, PDF file, 0.5 MB. Copyright © 2019 Xie et al. 2019 Xie et al. This content is distributed under the terms of the Creative Commons Attribution 4.0 International license.

Together, these results indicate that viral material is massively transferred from infected T cells to iDCs and OCs through heterotypic cell-to-cell fusion and that this cell fusion transfer is restricted to macrophage-tropic viral strains. In contrast, both macrophage- and lymphotropic viral strains can be captured by iDCs and mDCs from infected T cells without cell fusion. In addition, both NLAD8 and NL4.3 viral strains could be captured by purified blood monocytes without cell fusion with infected T cells after 6 h of coculture (Fig. S4B and C). Similarly, cell-to-cell transfer between infected T cells was observed across the so-called “virological synapse,” but no fusion was observed since almost all Gag^+^ target T cells contained only one nucleus (Fig. S4D and E), confirming that the process of cell fusion for HIV-1 transfer is specific to iDCs, OCs, and macrophages (25).

**FIG 3:**
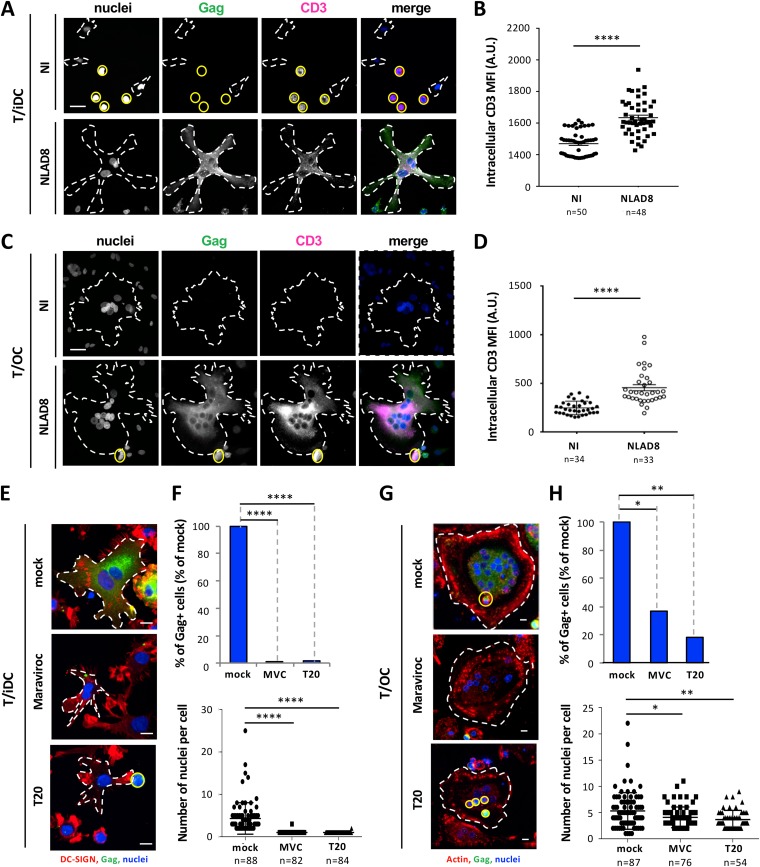
Cell fusion between infected T cells and iDCs or OCs by a viral-envelope-dependent mechanism. (A to D) NLAD8-infected Jurkat cells were cocultured for 6 h with iDCs (A and B) or OCs (C and D). Cells were then stained with anti-CD3, anti-Gag, and DAPI (nuclei) and analyzed by confocal microscopy. (A and C) Representative images of intracellular CD3 staining are shown (scale bars, 25 μm), and T cells and myeloid cells (OCs or iDCs) are indicated by plain yellow and dashed white lines, respectively. (B and D) Intracellular CD3 mean fluorescence intensities (MFI) were quantified as indicated in Materials and Methods. Each dot corresponds to 1 cell, and the number of cells analyzed is indicated (n). Horizontal bars represent means ± 1 SEM. Statistical significance was determined with the Mann-Whitney U-test (****, *P* < 0.0001). (E to H) NLAD8-infected Jurkat cells were pretreated or not (mock) with Maraviroc (MVC) or T20 for 1 h and cocultured with iDCs (E and F) or OCs (G and H) for 6 h in the presence of the inhibitor. Cells were then stained with DRAQ5 (nuclei) and anti-Gag, in combination with anti-DC-SIGN or phalloidin (actin), and analyzed by confocal microscopy (scale bar, 10 μm). (E and G) T cells and myeloid cells (iDCs or OCs) are indicated by plain yellow and dashed white lines, respectively. (F and H) The percentage of Gag^+^ iDCs or OCs (upper panels) and the number of nuclei per iDC or OC (lower panels) were determined from images of at least 50 cells. Each dot corresponds to 1 cell, and the number of cells analyzed is indicated (n). Horizontal bars represent means ± 1 SEM. Statistical significance was determined with the Mann-Whitney U-test (*, *P* < 0.05; **, *P* < 0.01; ****, *P* < 0.0001). The results shown are representative of at least 3 independent experiments performed with iDCs and OCs from 3 different donors. NI, noninfected Jurkat cells were cocultured with iDCs or OCs.

To confirm that transfer of macrophage-tropic viruses to iDCs and OCs was related to cell fusion with infected T cells, we checked that the Gag^+^ MGCs, formed upon coculture, expressed specific T cell markers. Jurkat or primary CD4 T cells were infected with macrophage-tropic virus strains (i.e., NLAD8, YU2, or JRFL) and then cocultured with iDCs or OCs for 6 h before staining for the CD3 and CD2 T-cell markers in addition to Gag was performed. As shown in Fig. 3A to D (see also Fig. S5A and B), the newly formed MGCs acquired the T cell CD3 and CD2 markers resulting from plasma membrane and cytoplasmic exchanges between infected T cells and iDCs/OCs through the cell fusion process. Finally, this cell fusion between infected T cells and OCs or iDCs was strongly inhibited by the CCR5 antagonist maraviroc (MVC) and the fusion inhibitor T20 (Fig. 3E to H), showing that this heterotypic cell fusion is envelope dependent and mediated by viral envelope-receptor interactions.

10.1128/mBio.02457-19.5 FIG S5 Characterization of iDC-derived multinucleated giant cells. (A) NLAD8-infected Jurkat cells were cocultured for 6 h with iDCs and then stained with anti-CD2, anti-Gag, and DAPI (Nuclei) and analyzed by confocal microscopy (scale bar, 20 μm). Intracellular CD2 mean fluorescence intensities (MFI) were quantified as indicated in Materials and Methods for at least 50 iDCs (right panel). Each dot corresponds to 1 cell, and the number of cells analyzed is indicated (n). Horizontal bars represent the means ± 1 SEM. Statistical significance was determined with the Mann-Whitney U-test (****, *P* < 0.0001). (B) Primary CD4^+^ T cells infected with viral strain NLAD8, JRFL, or YU2 were cocultured for 6 h with iDCs and then stained with anti-CD3, anti-Gag, and DAPI (Nuclei) and analyzed by confocal microscopy (scale bar, 20 μm). Intracellular CD3 MFI levels were quantified as indicated in Materials and Methods for at least 50 iDCs (right panel). Each dot corresponds to 1 cell, and the number of cells analyzed is indicated (n). Horizontal bars represent means ± 1 SEM. Statistical significance was determined with the Mann-Whitney U-test (****, *P* < 0.0001). (C) NLAD8-infected Jurkat cells were cocultured with iDCs for 24 h, and 1, 2, or 4 extensive washes were then performed with PBS in order to eliminate nonadherent cells. The remaining adherent cells were fixed, permeabilized, and stained with anti-Gag, anti-DC-SIGN, and DAPI (Nuclei) and analyzed by confocal microscopy (scale bar, 10 μm). The percentages of T cell removal before (No wash) or after 1, 2, or 4 washes were determined by quantification of the DC-SIGN(-) (T cell nuclei; green) or DC-SIGN(+) (iDC nuclei; blue) levels deduced from images of at least 50 cells (right panel). The results shown are representative of 4 independent experiments performed with iDCs from 4 different donors. NI, noninfected Jurkat cells were cocultured with iDCs. Download FIG S5, PDF file, 0.4 MB. Copyright © 2019 Xie et al. 2019 Xie et al. This content is distributed under the terms of the Creative Commons Attribution 4.0 International license.

### Virus dissemination through homotypic cell fusion between dendritic cells.

**FIG 4:**
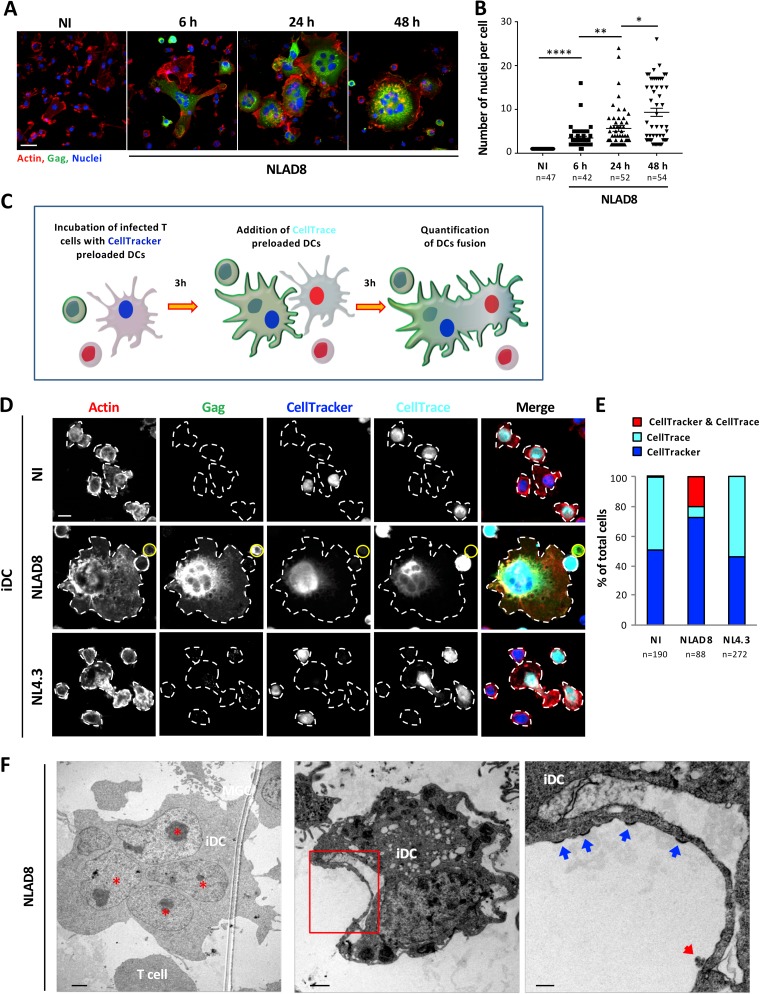
Virus dissemination between iDCs by homotypic cell fusion. (A and B) NLAD8-infected Jurkat cells were cocultured with iDCs for 6, 24, or 48 h and then stained with phalloidin (actin), anti-Gag, and DAPI (Nuclei) and analyzed by confocal microscopy (A; scale bar, 25 μm). (B) The number of nuclei per iDC was determined from images of at least 40 cells. Each dot corresponds to 1 cell, and the number of cells analyzed is indicated (n). Horizontal bars represent means ± 1 SEM. Statistical significance was determined with the Mann-Whitney U-test (*, *P* < 0.05; **, *P* < 0.01; ****, *P* < 0.0001). The results shown are representative of at least 3 independent experiments performed with iDCs from 3 different donors. NI, noninfected Jurkat cells were cocultured with iDCs. (C) Experimental protocol for homotypic cell fusion experiment. (D) NLAD8- or NL4.3-infected Jurkat cells were cocultured with iDCs prelabeled with CellTracker for 3 h, and autologous iDCs prelabeled with CellTrace were added and cultured for an additional 3 h. Cells were then stained with anti-Gag and phalloidin (actin) and analyzed by confocal microscopy (scale bar, 10 μm). (E) The number of single-positive CellTrace(+) and CellTracker(+) and of double-positive CellTrace(+)/CellTracker(+) iDCs was determined from images, and the results are expressed as the percentages of single- and double-positive iDCs. The number of cells analyzed is indicated (n). NI, noninfected Jurkat cells were cocultured with iDCs. (F) NLAD8-infected Jurkat cells were cocultured for 6 h with iDCs, and cells were then fixed and dehydrated. Ultrathin sections were cut, stained, and observed with a transmission electron microscope. A multinucleated iDC with nuclei (indicated by red stars) is shown (left image; scale bar, 2 μm). The right image (scale bar, 200 nm) corresponds to higher magnification of the area indicated in red on the central image (scale bar, 1 μm). Assembling and budding viruses are indicated by blue arrows, while mature virion is indicated by a red arrow. The results shown are representative of 3 independent experiments performed with iDCs from 3 donors.

Since we had observed that about half of the newly formed Gag^+^ lymphocyte/iDC fused cells contained 3 or more nuclei, including at least 2 CellTracker-negative nuclei (Fig. 2A; see also Fig. S2A and S3), we investigated whether lymphocyte/iDC fused cells were able to fuse with surrounding uninfected iDCs for virus dissemination. We first quantified the number of nuclei in iDC targets after 6, 24, or 48 h of coculture with NLAD8-infected T cells. After 6 h of coculture, about 95% of Gag^+^ iDCs contained more than 2 nuclei, with an average number of nuclei per cell of 3.5, which increased to 5.7 and 9.3 nuclei after 24 and 48 h of coculture, respectively (Fig. 4A and B). Interestingly, we observed that after 24 h of coculture with infected T cells, the newly formed Gag^+^ lymphocyte/iDC fused cells acquired adhesion properties demonstrated by immunofluorescence analysis performed directly on coverslips, since more than 98% of T cells were removed from the coculture after 4 extensive washes, whereas iDCs cocultured with uninfected T cells were still nonadherent and were mostly removed by a single wash (Fig. S5C).

To demonstrate the homotypic fusion between lymphocyte/iDC fused cells and surrounding uninfected iDCs, NLAD8-infected T cells were cocultured with iDCs preloaded with CellTracker for 3 h, and autologous iDCs preloaded with the CellTrace dye were then added for an additional 3 h before staining for intracellular Gag was performed as schematized on Fig. 4C. When iDCs were initially cocultured with uninfected or NL4.3-infected T cells, iDCs stained with either CellTracker or CellTrace and did not show diffuse cytoplasmic Gag staining (Fig. 4D and E). In contrast, when iDCs were cocultured with NLAD8-infected T cells, we observed that about 20% of the Gag^+^ multinucleated iDCs were stained with both CellTracker and CellTrace dyes (Fig. 4D and E). These results indicate that the first heterotypic fusion step between infected T cells and CellTracker-positive iDCs is followed by a second homotypic fusion with uninfected CellTrace-positive iDCs, leading to the formation of Gag^+^ multinucleated giant iDCs.

We also used electron microscopy to visualize multinucleated giant iDCs after 6 h of coculture with NLAD8-infected T cells (Fig. 4F, left image). More interestingly, electron-dense material reminiscent of Gag assembly corresponding to virus buds (blue arrows), as well as mature virions (red arrows), were observed protruding from the plasma membrane of iDCs (Fig. 4F, central and right images), indicating that virus assembly and budding took place at the cell surface of iDCs after only 6 h of coculture with infected T cells. These observations also confirm plasma membrane exchanges and the transfer of Gag material between infected T cells and iDC targets.

### Virus spreading in myeloid cells through cell fusion escapes SAMHD1 restriction.

**FIG 5:**
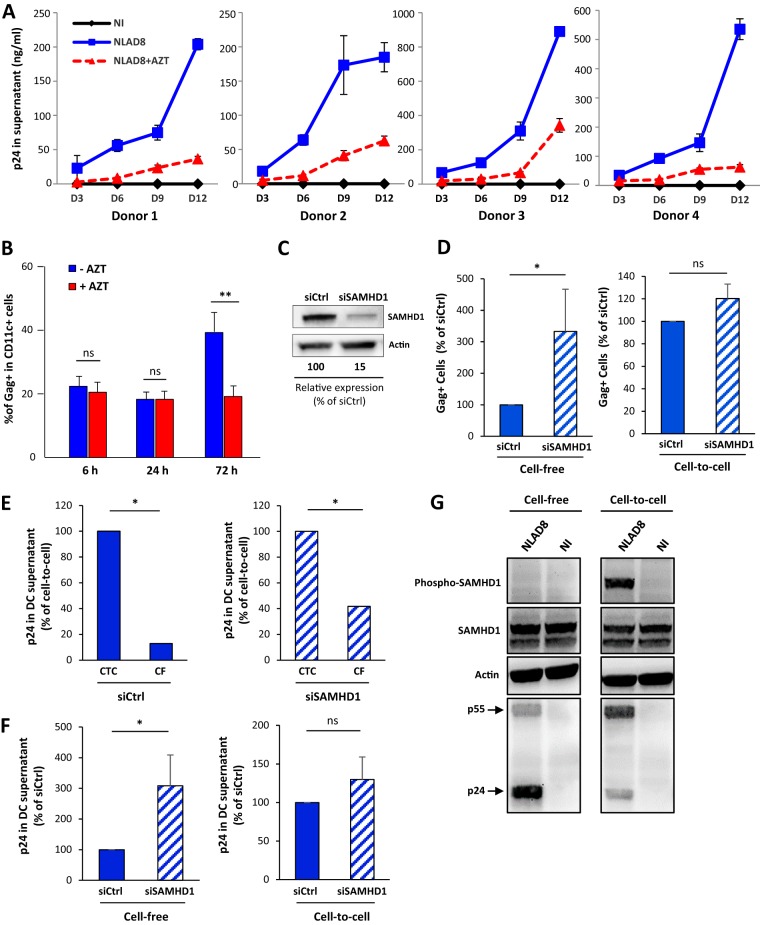
Productive infection of iDCs by cell-to-cell fusion with infected T cells escapes SAMHD1 restriction. (A) NLAD8-infected Jurkat cells were cocultured for 24 h with iDCs derived from 4 different donors with or without AZT. T cells were then eliminated by extensive washes as described previously, and iDCs were cultured with or without AZT for different period of time. Cell culture supernatants from iDCs were collected after 3, 6, 9, or 12 days, and viral p24 was quantified. Each experiment was performed in duplicate. NI, noninfected Jurkat cells were cocultured with iDCs. (B) NLAD8-infected Jurkat cells were cocultured for 6, 24, or 72 h with iDCs with or without AZT, and the percentage of Gag^+^/CD11c^+^ iDCs was then evaluated by flow cytometry. The results represent means of 3 independent experiments performed with iDCs of 3 different donors. Error bars represent 1 SEM. Statistical significance was determined using the Mann-Whitney U-test (ns, *P* > 0.05; **, *P* < 0.01). (C) iDCs were transfected with siRNA targeting SAMHD1 (siSAMHD1) or scrambled siRNA (siCtrl) used as a control. Lysates from transfected cells were analyzed by Western blotting using anti-SAMHD1 and anti-β-actin antibodies. The band intensities were quantified using NIH Image software, and the signal measured in siRNA-transfected cells was normalized to the signal obtained for actin. The indicated values are expressed as percentages of the signal intensity relative to siCtrl (100%) and are representative of 3 independent experiments. (D to F) iDCs transfected with siCtrl (blue bars) or siSAMHD1 (hatched bars) were infected by cell-free NLAD8 viruses produced by Jurkat cells (CF) or by coculture with NLAD8-infected Jurkat cells (CTC) for 24 h. (D) The percentage of Gag^+^/DC-SIGN^+^ iDCs was evaluated by flow cytometry directly after 24 h of infection with CF or CTC. The results are expressed as the percentage of Gag^+^ iDCs relative to that obtained with siCtrl-transfected iDCs (100%). (E and F) Quantification of p24 released in cell culture supernatants from siRNA-transfected iDCs infected by CF or CTC for 24 h and then cultured for 6 more days. The results are expressed as the percentage of p24 released relative to that released by CTC infection (E), or as the percentage of p24 released relative to that released by siCtrl-transfected iDCs (F). Results represent means of 4 independent experiments performed with iDCs from 4 different donors. Error bars represent 1 SEM. Statistical significance was determined using the Mann-Whitney U-test (ns, *P* > 0.05; *, *P* < 0.05; **, *P* < 0.01). (G) MDMs were infected by cell-free NLAD8 viruses produced by CD4^+^ primary T cells (Cell-free, left panels) or by coculture with NLAD8-infected primary T cells (Cell-to-cell, right panels) for 24 h. Cell lysates were then analyzed by Western blotting using anti-SAMHD1, anti-phospho-SAMHD1, anti-HIV-1 p24, and anti-β-actin antibodies.

Finally, we investigated whether this virus cell-to-cell transfer and dissemination through cell fusion resulted in the productive infection of iDCs. We took advantage of the acquisition of adherence properties of the MGCs formed through the two-step cell fusion process for elimination of infected T cells (see Fig. S5C) in order to monitor virus production generated by iDC-derived MGCs after virus transfer. Infected T cells and iDCs were cocultured for 24 h before elimination of T cells by extensive washes, and viral p24 released by iDC-derived MGCs was monitored every 3 days. Gag^+^ multinucleated giant iDCs containing many nuclei were still alive 9 days after T cell removal (Fig. S6A) and produced high levels of p24 (Fig. 5A, blue lines). Since iDCs have the ability to capture and release virus particles without virus replication (34, 35) (see Fig. S4A), we analyzed the effect of the reverse transcription (RT) inhibitor zidovudine (AZT) on virus dissemination in iDCs and on p24 release. When iDCs were treated with AZT during and after coculture with infected T cells, virus dissemination evaluated by the percentage of Gag^+^ iDCs (Fig. 5B) and levels of virus production (Fig. 5A, red lines) were significantly reduced, without affecting the initial step of virus cell-to-cell transfer from infected T cells (Fig. S6B and C). These results show that virus-mediated cell-to-cell fusion of infected T cells with iDCs is a very efficient process for virus dissemination through the formation of highly virus-productive MGCs, suggesting that this cell fusion process may bypass the restriction imposed by host cell restriction factors such as SAMHD1 expressed in iDCs. To test this hypothesis, specific small interfering RNAs (siRNA) were transfected in iDCs or MDMs, leading to a net decrease in SAMHD1 expression (Fig. 5C; see also Fig. S7A), and virus transfer and dissemination was evaluated in SAMHD1-depleted myeloid cells infected by NLAD8 cell-free (CF) viruses or after 24 h of coculture with infected T cells (CTC). As expected, virus transfer, evidenced through analysis of the percentage of Gag^+^ iDCs, was significantly increased when SAMHD1-depleted iDCs or MDMs were infected by cell-free viruses (Fig. 5D, left panel; see also Fig. S7B, left panel). In contrast, no difference in the percentage of Gag^+^ iDCs was observed when siCtrl- and siSAMHD1-transfected iDCs or MDMs were cocultured with infected T cells (Fig. 5D, right panel; see also Fig. S7B, right panel). Similarly, virus dissemination assessed 6 days later by p24 quantification in the cell culture supernatant was still significantly higher when iDCs or MDMs were infected by CTC viruses than when were infected by CF viruses (Fig. 5E; see also Fig. S7C), and SAMHD1 depletion did not show an impact on the high level of virus production observed when iDCs or MDMs were initially cocultured with infected T cells (Fig. 5F; see also Fig. S7D). These results demonstrate that the restriction of virus replication imposed by SAMHD1 expression through cell-free virus infection of myeloid cells is bypassed through formation of myeloid cell-derived MGCs able to produce high levels of virus particles.

10.1128/mBio.02457-19.6 FIG S6 Survival of iDC-derived MGCs and effect of AZT on MGC formation. (A) NLAD8-infected Jurkat cells were cocultured with iDCs for 24 h, and nonadherent cells were eliminated by 4 washes with PBS. Adherent cells were then cultured for 3 days (D3), 6 days (D6), or 9 days (D9) before fixation, permeabilization, and staining were performed with anti-Gag, phalloidin (actin)and DAPI (Nuclei) and analyzed by confocal microscopy (scale bar, 10 μm). (B and C) NLAD8-infected Jurkat cells were cocultured for 24 h with iDCs with or without AZT. Cells were then stained with anti-Gag, anti-DC-SIGN, and DRAQ5 (nuclei). Cells were analyzed by confocal microscopy (B, scale bar, 10 μm). (C) The number of nuclei per iDC was determined from images. Each dot corresponds to 1 cell, and the number of cells analyzed is indicated (n). Horizontal bars represent means ± 1 SEM. Statistical significance was determined with the Mann-Whitney U-test (ns, *P* > 0.05). The results shown are representative of 4 independent experiments performed with iDCs from 4 donors. Download FIG S6, PDF file, 0.3 MB. Copyright © 2019 Xie et al. 2019 Xie et al. This content is distributed under the terms of the Creative Commons Attribution 4.0 International license.

10.1128/mBio.02457-19.7 FIG S7 Productive infection of MDMs by cell-to-cell fusion with infected T cells escapes SAMHD1 restriction. (A) MDMs were transfected with siRNA targeting SAMHD1 (siSAMHD1) or scrambled siRNA (siCtrl) used as a control. Lysates from transfected cells were analyzed by Western blotting using anti-SAMHD1 and anti-β-actin antibodies. The band intensities were quantified using NIH Image software, and the signal measured in siRNA-transfected cells was normalized to the signal obtained for actin. The indicated values are expressed as percentages of the signal intensity relative to siCtrl (100%). (B to D) MDMs transfected with siCtrl (blue bars) or siSAMHD1 (hatched bars) were infected by cell-free NLAD8 viruses produced by Jurkat cells (CF) or by coculture with NLAD8-infected Jurkat cells (CTC) for 24 h. As shown in panel B, the percentage of Gag^+^ MDMs was evaluated by flow cytometry directly after 24 h of infection with CF or CTC. The results are expressed as the percentage of Gag^+^ MDMs relative to that obtained with siCtrl-transfected MDMs (100%). In panels C and D, data represent results of quantification of p24 released in cell culture supernatants from siRNA-transfected MDMs infected by CF or CTC for 24 h and then cultured for 6 more days. The results are expressed as the percentage of p24 released relative to that released by CTC infection (C) or as the percentage of p24 released relative to that released by siCtrl-transfected iDCs (D). (E) MDMs were infected by cell-free NLAD8 viruses produced by Jurkat cells (Cell-free; left panels) or by coculture with NLAD8-infected Jurkat (Cell-to-cell; right panels) for 24 h. Cell lysates were then analyzed by Western blotting using anti-SAMHD1, anti-phospho-SAMHD1, anti-HIV-1 p24, and anti-β-actin antibodies. Download FIG S7, PDF file, 0.1 MB. Copyright © 2019 Xie et al. 2019 Xie et al. This content is distributed under the terms of the Creative Commons Attribution 4.0 International license.

Since only the dephosphorylated form of SAMHD1 is able to restrict HIV-1 replication (36–38), we investigated further the SAMHD1 restriction escape mechanism by analyzing SAMHD1 expression and phosphorylation in myeloid cells infected by cell-free viruses or by cell fusion with infected T cells. As usual, NLAD8-infected T cells were cocultured for 24 h with MDMs before cell lysis and Western blotting of total and phosphorylated SAMHD1 in MGCs formed upon cell fusion with infected T cells (Fig. 5G; see also Fig. S7E). As expected, SAMHD1 was expressed in its active dephosphorylated form for HIV-1 restriction when MDMs were infected with cell-free viruses (Fig. 5G; see also Fig. S7E, left panels). In sharp contrast, most of the SAMHD1 protein was phosphorylated when MDM targets were infected by cell-to-cell fusion with infected T cells (Fig. 5G; see also Fig. S7E, right panels). These findings show that the two-step cell fusion process leading to MGC formation results in phosphorylation of SAMHD1, allowing efficient HIV-1 replication and spreading in myeloid cells.

## DISCUSSION

In this report, we reveal that HIV-1 uses a specific and common two-step cell fusion process for cell-to-cell transfer and dissemination from infected CD4^+^ T cells to target cells of the myeloid lineage, including macrophages, OCs, and iDCs, but not monocytes and mDCs. This two-step cell fusion mechanism bypasses the SAMHD1-related restriction of HIV-1 replication observed in these myeloid cells with cell-free viruses and may be responsible for virus spreading in these cell targets *in vivo*, as observed in host lymphoid and nonlymphoid tissues, including the central nervous system and bones (39–43). In agreement, it was reported that myeloid cells from spleen and lymph nodes of SIV-infected macaques contain T cell markers and viral RNA and DNA originating from infected T cells (44, 45).

We have shown here that virus cell-to-cell transfer from infected T cells is mediated first by heterotypic cell fusion with macrophages, OCs, or iDCs and then by virus dissemination through a second homotypic fusion with uninfected myeloid cells, leading to the formation of highly virus-productive MGCs. This two-step cell fusion process is specific for these myeloid cell targets, since HIV-1 cell-to-cell transfer between CD4^+^ T cells is mediated mainly by the formation of the virological synapse without cell fusion between virus donor and target T cells (46, 47) (and see Fig. S4D and E). Similarly, we did not detect cell fusion for cell-to-cell transfer of HIV-1 from infected T cells to CD4^+^ monocytes or mDCs, suggesting that the process of cell fusion between T cells and iDCs, OCs, or macrophages is finely regulated. Previous studies using cell-free viruses proposed that the lower sensitivity of monocytes and mDCs to HIV-1 was related to the low expression of the CCR5 coreceptor at the cell surface of these cell types (48, 49), and monocytes and mDCs indeed express low levels of CCR5 compared to iDCs and differentiated macrophages (Fig. S1E and data not shown). In addition, it was suggested that some cell surface molecules, such as tetraspanin proteins, might have a negative regulatory role in cell fusion and formation of syncytia between infected and target T cells after formation of the virological synapse (50). While our data show that interactions between the viral envelope and the CD4 receptor and CCR5 coreceptor are required for the cell fusion process that leads to formation of HIV-1-infected MGCs originating from macrophages, OCs, or iDCs, it would be interesting to investigate the potential role of some cell surface proteins (51), such as tetraspanins, in the mechanism of HIV-1-mediated cell-to-cell fusion between lymphoid and myeloid cells described here. Similarly, we cannot exclude the possibility that some signaling pathways and effectors involved in programming of myeloid cells into a fusion-competent state could also participate in the two-step cell fusion process for HIV-1 cell-to-cell spreading in myeloid cells. Depending of the cytokine environment, it was established that cells of the myeloid lineage have the propensity to mediate homotypic cell fusion leading to the formation of multinucleated OCs and MGCs under both physiological and pathological conditions (52, 53). While the formation in bones of functional multinucleated OCs is mainly orchestrated by the receptor activator of NF-κB ligand (RANKL) cytokine, alternative activation of macrophages by interleukin-4 (IL-4) and IL-13 can promote homotypic macrophage fusion and MGC formation, as observed in some inflammatory disorders (53). Therefore, it would be interesting to explore the contribution of cellular pathways mobilized by these cytokines in both cell fusion steps allowing HIV-1 cell-to-cell spreading in myeloid cells.

HIV-1 transfer from infected T cells to myeloid cells through cell fusion is a fast and very efficient process leading to transfer of viral material toward 20% to 40% of the myeloid target cells after only 6 h of coculture. This process leads to virus spreading in iDCs, OCs (data not shown), or macrophages (25) through the formation of MGCs able to produce large amount of viral particles over several days and weeks. In contrast, many reports indicate that these myeloid cells are poorly infected by cell-free viruses, because virus replication is limited by the high expression of host cell restriction factors, such as SAMHD1 (54). When noncycling myeloid target cells are infected with cell-free viruses, SAMHD1 is expressed in its dephosphorylated active form and is able to restrict HIV-1 replication (36–38). In contrast, our findings show that the two-step cell fusion process results in phosphorylation of SAMHD1, allowing efficient virus spreading in iDCs and macrophages. Interestingly, Puigdomènech et al. (55) previously suggested that HIV-1 cell-to-cell transmission from infected T cells to DCs was restricted by SAMHD1 expression. Even if similar experimental systems and viral strains were used in the two studies, the discrepancy with our findings can be explained by the gating strategy used to analyze virus dissemination by flow cytometry, since the Gag^+^ iDC-derived MGCs formed through cell fusion during coculture with infected T cells were certainly excluded from the analysis performed by Puigdomènech and colleagues (55). In addition, they did not perform fluorescence microscopy analysis to visualize and study MGC formation through cell fusion of infected T cells with iDC targets. In contrast, our data demonstrate that this cell-to-cell fusion process supports high levels of virus production by iDC-, OC-, and macrophage-derived MGCs, during which SAMHD1 is expressed in its inactive phosphorylated form, suggesting that the cyclin/cyclin-dependent kinase (CDK) complex involved in SAMHD1 phosphorylation is expressed in MGCs. While further investigations are thus needed to elucidate this intriguing mechanism of SAMHD1 phosphorylation in MGCs, we can speculate that the cyclin/CDK complex could be transferred from activated infected T cells through the first heterotypic cell fusion and through delivery of the cellular content to the myeloid target cell. In addition, the high levels of deoxynucleoside triphosphates (dNTPs) transferred from proliferative infected T cells could also help to overcome the restriction activity of SAMHD1 expressed in myeloid cells. Alternatively, an attractive hypothesis could be related to the maintenance of transcriptionally active T cell nucleus after the initial fusion between infected T cells and myeloid target cells (L. Bracq and S. Benichou, unpublished observations). Thus, transfer of the T cell nucleus already containing integrated proviral DNA may allow bypass of the reverse transcription step restricted by SAMHD1, leading to efficient SAMHD1-independent viral expression by the newly fused cells formed through cell fusion between infected T cells and myeloid cells.

While the physiological formation of OCs results from cell-to-cell fusion of monocyte/macrophages (52, 53), we show that multinucleated OCs are also able to fuse with HIV-1-infected T cells for virus dissemination in these cell targets. Similarly, iDC targets can fuse with infected T cells for HIV-1 cell-to-cell transfer followed by a second step of homotypic fusion with uninfected iDCs leading to the formation of MGCs. Interestingly, it has been shown that DCs were able to fuse together and to acquire new phenotypical properties under inflammatory conditions (56) and in granulomatous diseases (57) or proliferating disorders such as Langerhans cell histiocytosis (58). In agreement with our results, heterotypic fusion of DCs with HIV-1-infected T cells was already suggested 20 years ago by the group of R. M. Steinman (7, 59). That group and others reported the presence of HIV-1-infected multinucleated syncytia expressing specific markers of both DCs and T cells at the surface of the nasopharyngeal tonsils and the adenoid and parotid gland of HIV-1-infected patients (7, 60–62), but the mechanisms related to these *in vivo* observations were not further investigated. Our study demonstrated that the cell fusion process for HIV-1 cell-to-cell transfer from T cells to iDCs is responsible for the formation of the multinucleated infected DCs observed in infected patients.

In summary, the novel and very efficient two-step cell fusion mechanism we report for virus cell-to-cell spreading is specific to iDC, OC, and macrophage target cells of HIV-1. Infected T cells first fuse with these myeloid cells for massive viral transfer, followed by a second step of homotypic fusion with uninfected myeloid cells for virus dissemination. Both steps are dependent on the viral envelope and seem restricted to macrophage-tropic viral strains. While HIV-1 infection of these myeloid cells by cell-free viruses is known to be strongly restricted (28, 63), this new mechanism of virus spreading in myeloid cells by cell fusion escapes the restriction imposed by SAMHD1 and leads to the formation of highly virus-productive MGCs reminiscent of the infected MGCs originating from myeloid cells observed *in vivo* in infected patients and SIV-infected macaques. These *in vivo* observations support the idea of the importance of the mechanisms revealed here, which contribute to a better understanding of virus cell-to-cell dissemination in myeloid cells and their roles in HIV-1 pathogenesis, including cell-associated virus sexual transmission, virus spreading, and establishment of long-lived viral tissue reservoirs.

## MATERIALS AND METHODS

### Plasmids and reagents.

The proviral plasmids pNL4.3 and pNLAD8 were obtained from the AIDS Research and Reference Reagent Program, Division of AIDS, NIAID, while the plasmid encoding the VSV-G envelope glycoprotein (pVSVg) has been described previously (64). The following antibodies were used: phycoerythrin (PE)- or fluorescein isothiocyanate (FITC)-conjugated anti-CD11b (clone ICRF44; BD Biosciences), PE-Cy7-conjugated anti-CD11b (clone ICRF44; Biolegend), RD1- or FITC-conjugated anti-Gag (clone KC57; Beckman Coulter), anti-CD4 (clone Leu3a; Biolegend), anti-CD3 (clone UCHT1; Biolegend), anti-DC-SIGN (clone 120507; R&D Systems), FITC-conjugated anti-CD11c (clone B-ly6; BD Biosciences), anti-CD14 (clone M5E2; BD Biosciences), allophycocyanin (APC)-conjugated anti-CD83 (clone HB15; Miltenyi), Alexa Fluor 647-conjugated anti-CCR5 (clone HEK/1/85a; Biolegend), PE-conjugated anti-CXCR4 (clone 12G5; Biolegend), anti-CD2 (clone TS2/18; a gift from Andres Alcover, Paris, France [65]), and Alexa Fluor 647- or Alexa Fluor 555-conjugated phalloidin (Life Technologies). The following reagents were obtained from the AIDS Research and Reference Reagent Program, Division of AIDS, NIAID: HIV-1 CAp24 hybridoma (183-H12-5C), HIV-IG, Maraviroc, T20, and AZT.

### Cell culture.

HEK293T and Jurkat cell lines were obtained from the ATCC. HEK293T cells were maintained in Dulbecco minimal essential medium (DMEM) supplemented with 10% heat-inactivated fetal calf serum (FCS), 100 IU of penicillin/ml, and 100 μg of streptomycin/ml (ATB; Invitrogen). Jurkat cells (clone J77) were maintained in RPMI 1640 complete culture medium supplemented with 10% FCS and ATB. Peripheral blood mononuclear cells (PBMCs) were isolated from blood of healthy anonymous donors by density gradient sedimentation using Histopaque (Sigma), and monocytes were purified using a CD14-positive selection kit (CD14 microbeads; Miltenyi) according to manufacturer’s guidelines. Blood samples from anonymous healthy donors were purchased from Etablissement Français du Sang Paris-Saint-Antoine-Crozatier, Paris, France, or from Changhai Hospital, Shanghai, China. The monocytes were differentiated into macrophages for 8 days in RPMI 1640 culture medium supplemented with 20% FCS, ATB, and 25 ng/ml of granulocyte-macrophage colony-stimulating factor (GM-CSF) and macrophage colony-stimulating factor (M-CSF) (Miltenyi). Monocytes were differentiated into osteoclasts for 4 days in RPMI 1640 culture medium supplemented with 20% FCS, ATB, 50 ng/ml of M-CSF, and 30 ng/ml of RANKL and were then cultured for another 6 days with 25 ng/ml of M-CSF and 100 ng/ml of RANKL, allowing complete maturation. The markers and functionality (bone degradation) of OCs were analyzed as previously described (22). In addition, RNA levels of specific OC markers were assessed by quantitative PCR (qPCR) analysis as described by Souriant et al. (66). Finally, monocytes were differentiated into iDCs for 5 days in RPMI 1640 culture medium supplemented with 20% FCS, ATB, and 50 ng/ml of GM-CSF and IL-4 (Miltenyi). Maturation of DCs was performed using 1 μg/ml of LPS (Escherichia coli O55:B5; Sigma-Aldrich) for 48 h. Human primary CD4^+^ T cells were isolated from PBMCs by density gradient sedimentation using Histopaque and then purified by negative selection (CD4^+^ T cell isolation kit; Miltenyi) following the manufacturer’s recommendations. CD4^+^ T cells were activated for 3 days in RPMI medium containing 20% FBS, interleukin-2 (IL-2; Miltenyi) at 10 μg/ml, and phytohemagglutinin-P at 5 μg/ml (PHA-P; Sigma-Aldrich). After activation, CD4^+^ T cells were kept in RPMI medium supplemented with 20% FBS and IL-2. All cells were grown at 37°C in 5% CO_2_.

### Viral production, titration, and infection.

Replication-competent HIV-1 NL4.3 and NLAD8 strains were produced in HEK293T cells by cotransfection of the proviral plasmid in combination with pVSVg using a previously described calcium phosphate precipitation technique (50). The amounts of p24 produced were determined by enzyme-linked immunosorbent assay (ELISA; Innogenetics). Viral titers were determined using Jurkat cells and flow cytometry (Accuri C6; BD Biosciences) as described previously (64).

### Cell-to-cell viral transfer and dissemination.

To study virus transfer from infected Jurkat or primary CD4^+^ T cells to myeloid cells, T cells were infected using a multiplicity of infection (MOI) of 0.5 for 16 h and were then washed and cultured for another 24 h. After washing, T cells were cocultured for 3 to 72 h at a 1:1 ratio with MDMs, OCs, or iDCs seeded at a density of 0.5 × 10^6^, 1 × 10^6^, or 0.4 × 10^6^ cells/well, respectively. Where indicated, iDCs or MDMs were transfected with a mix of two different siRNAs targeting SAMHD1 at a concentration of 100 nM each (Dharmacon, catalog no. L-013950-09 and L-013950-10) using Hiperfect transfection reagent (Qiagen) accordingly to the recommendations of the manufacturer. At 24 h after transfection, a second round of transfection with siRNA was performed using the same protocol followed by coculture with infected T cells for 6 h. To remove T cells after coculture with myeloid cells, 4 extensive washes with phosphate-buffered saline (PBS) were performed. MDMs, OCs, or DCs were harvested or cultured for several days and then collected. Cells were then surface stained using anti-CD11b, anti-CD11c, anti-DC-SIGN, anti-CD2, or anti-CD3 antibodies and were then fixed with 4% paraformaldehyde (PFA) and permeabilized and stained using anti-Gag (KC57; 1/500) and permeabilization buffer (Beckman Coulter). The percentages of Gag^+^ cells among CD11b^+^, CD11c^+^, or DC-SIGN^+^ cells corresponding to the MDM, OC, or DC population, respectively, were determined by flow cytometry or by fluorescence microscopy as described previously (22, 25). To analyze viral production, cell culture supernatants of iDCs were collected, and the amount of p24 produced was determined by ELISA.

### Effect of inhibitors on viral transfer and dissemination in iDCs and OCs.

To analyze the effect of inhibitors on virus transfer, infected donor T cells and OC or iDC targets were pretreated for 1 h with T20 or Maraviroc at 10 μM and 10 μg/ml, respectively. Infected T cells were then cocultured for 6 h with OCs or iDCs. For OCs, T cells were eliminated by extensive washes, and the percentage of Gag^+^ OCs was determined by flow cytometry or fluorescence microscopy as described previously (20, 24). For iDCs, mixtures of T cells and iDCs were collected and the percentage of Gag^+^ CD11c^+^ iDCs was determined by flow cytometry. Results were expressed as the percentage of Gag^+^ OCs or iDCs relative to that determined without inhibitors. To show that virus transfer from infected T cells to iDCs led to productive infection, iDC targets were pretreated with AZT (10 μM) for 2 h prior to coculture for 6, 24, or 72 h with infected T cells. After removal of infected T cells by 4 extensive washings with PBS, the percentage of Gag^+^ CD11b^+^ iDCs was determined by flow cytometry directly after the 6, 24, or 72 h of coculture, while the viral p24 production from iDCs was determined by ELISA after 3, 6, 9, or 12 more days of culture in the presence of AZT.

### Fluorescence microscopy analysis.

To visualize cell contacts, virus transfer, and virus dissemination, Jurkat or primary CD4^+^ T cells infected with NLAD8 or NL4.3 as described above were preloaded with 5 μM of CellTracker CMAC (7-amino-4-chloromethylcoumarin) (Life Technologies) and cocultured for 6, 24, or 48 h with OCs (plated onto coverslips), DCs, monocytes, or CD4^+^ T cells. After coculture, OCs were directly fixed with 4% PFA while the mixture of DCs (or monocyte or T cell targets) and virus donor infected T cells was seeded on l-polylysine-coated coverslips (Sigma-Aldrich) before fixation. Cells were then blocked for 10 min in PBS containing 1% bovine serum albumin (BSA) and stained with 2 μM of DRAQ5 (eBioscience) for 20 min in PBS for staining of nuclei, followed by surface staining of DC-SIGN (1/100 dilution) for 1 h and incubation with Alexa Fluor 555-coupled secondary antibody, and then permeabilized and stained using KC57 FITC-conjugated antibody or phalloidin-Alexa Fluor 647 (Molecular Probes) diluted in permeabilization buffer for 1 h. Coverslips were then washed with PBS and mounted on slides using 10 μl of Fluoromount (Sigma) or Fluroromount containing DAPI (4′,6-diamidino-2-phenylindole) (Sigma-Aldrich). Images were acquired on a spinning disk (CSU-X1M1; Yokogawa)-equipped inverted microscope (DMI6000; Leica) and were then processed using Fiji software (ImageJ; NIH) (67). Quantitative image analysis was performed using Fiji by defining a region of interest using the actin or DC-SIGN staining and measuring the whole fluorescence intensity of the Gag staining, with respect to noninfected cells. To analyze fusion between infected T cells and OCs or iDCs, infected Jurkat or primary CD4^+^ T cells were cocultured for 6 or 24 h with myeloid cells. Cells were then fixed with 4% PFA and blocked in PBS containing 1% BSA. Anti-CD3 (10 μg/ml), anti-CD2 (1/200 dilution), anti-DC-SIGN, anti-Gag (KC57-FITC; 1/200 dilution), and F-actin (with fluorescent phalloidin) intracellular staining were done by incubating coverslips with the indicated primary antibody and phalloidin-Alexa Fluor 647 diluted in the permeabilization buffer. Coverslips were then rinsed with PBS-BSA and incubated for 1 h with the corresponding fluorescence-coupled secondary antibody. After 3 washes, coverslips were mounted on microscope slides using 10 μl of Fluoromount mounting medium containing DAPI. Cells were examined under an epifluorescence microscope (DeltaVision), and quantitative image analysis was performed using Fiji software by defining a region of interest using the F-actin staining and measuring the whole fluorescence intensity of the indicated marker (i.e., CD3 or CD2) in Gag^+^ cells, with respect to noninfected cells. To analyze the effect of inhibitors on fusion between T cells and OCs and iDCs, infected virus donor T cells, or OC and iDC targets were pretreated for 1 h with T20, Maraviroc, or AZT at 10 μM, 10 μg/ml, or 10 μM, respectively. Infected T cells were then cocultured for 6 h with OCs or iDCs in the presence of inhibitors. Cells were then fixed and stained as described previously using KC57 FITC-conjugated antibody and phalloidin-Alexa Fluor 647. Images were acquired and then processed as described above. To analyze fusion between iDCs, infected T cells were initially cocultured with iDCs preloaded with CellTracker CMAC for 3 h. Autologous iDCs prelabeled with CellTrace-Far-red were then added to the mixture of infected T cells and iDCs and cultured for an additional 3 h. The mixtures of iDCs and T cells were then seeded on polylysine coverslips, fixed with PFA 4%, stained with anti-Gag (KC57-FITC) and phalloidin-Alexa Fluor 555, and mounted with Fluoromount media. Images were acquired and processed as described above. To analyze viral transfer between T cells or between T cells and monocytes, infected T cells preloaded with CellTracker CMAC or CellTrace-Far-red were cocultured with unloaded T cells or monocytes for 6 h. The mixtures of cells were then collected and seeded on coverslips coated with polylysine; fixed with PFA 4%; permeabilized and stained for nuclei (DRAQ5), intracellular Gag (KC57-FITC), or phalloidin-Alexa Fluor 647; and mounted with Fluoromount media. Images were acquired and processed as described above. The number of nuclei per cell was determined from images of at least 50 cells followed by processing using Fiji as described above.

### Transmission electron microscopy analysis.

Jurkat cells infected with NLAD8 or NL4.3 as described above were cocultured for 6 h with iDCs. Cells were then collected and seeded on coverslips coated with polylysine. The coverslips were then fixed using glutaraldehyde 2.8% and 2% PFA for 20 min. After 2 washes in PBS, cells were dehydrated and embedded into epoxy (Electron Microscopy Sciences) as described previously (25). Ultrathin (90-nm) sections were cut with an ultramicrotome, stained with uranyl acetate and Reynold’s lead, and observed with a transmission electron microscope (JEOL 1011) as described previously (25). Acquisition was performed with a Gatan ES1000W charge-coupled-device (CCD) camera.

### Western blotting.

For analysis of the levels of expression of total and phosphorylated SAMHD1 in siRNA-transfected iDCs or MDMs, as well as in MDM-derived MGCs, cells (10^6^) were lysed in 100 μl of reducing Laemmli sample buffer (2×) supplemented with PhosSTOP (phosphatase inhibitor cocktail tablets; Sigma-Aldrich) and cOmplete (EDTA-free protease inhibitor cocktail; Sigma-Aldrich) and then boiled at 96°C for 10 min. Cell lysates were then resolved by SDS-PAGE as described previously (64) and analyzed by Western blotting using anti-SAMHD1 (Abcam; ab67820), anti-phospho-SAMHD1 (Cell Signaling; catalog no. 89930), anti-HIV-1 p24 (Abcam; ab9071), and anti-β-actin (Sigma-Aldrich; clone AC-15) antibodies.
