# Supplementary figures and images for: Cell-to-Cell Spreading of HIV-1 in Myeloid Target Cells Escapes SAMHD1 Restriction
Source: mBio. 2019 Nov 19;10(6):e02457-19. doi: 10.1128/mBio.02457-19 (PMC6867896; doi:10.1128/mBio.02457-19)

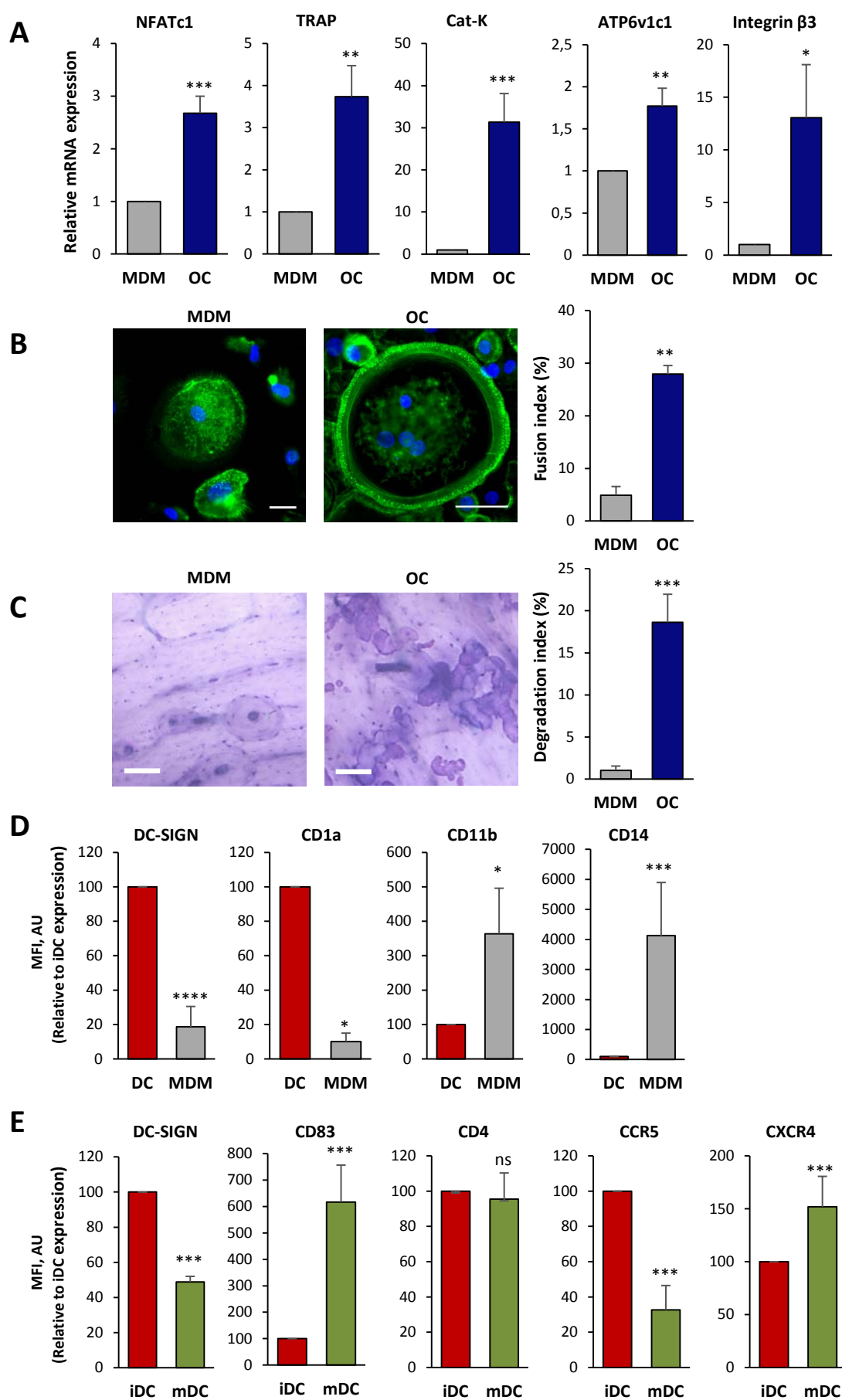

Supplementary Fig. S1

Supplement: FIG S1 [file mBio.02457-19-sf001.pdf]

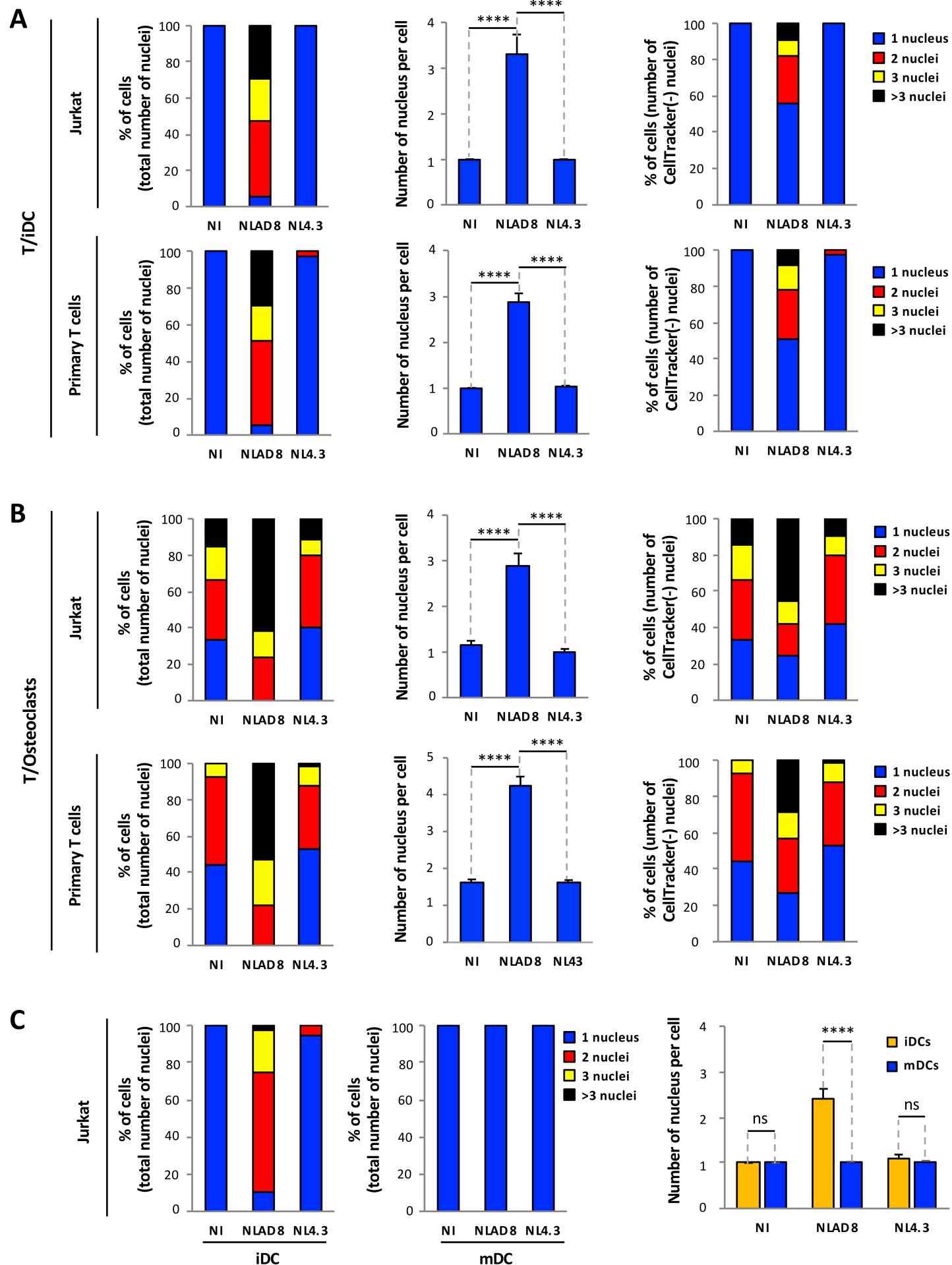

Supplementary Fig. S2

Supplement: FIG S2 [file mBio.02457-19-sf002.pdf]

**A**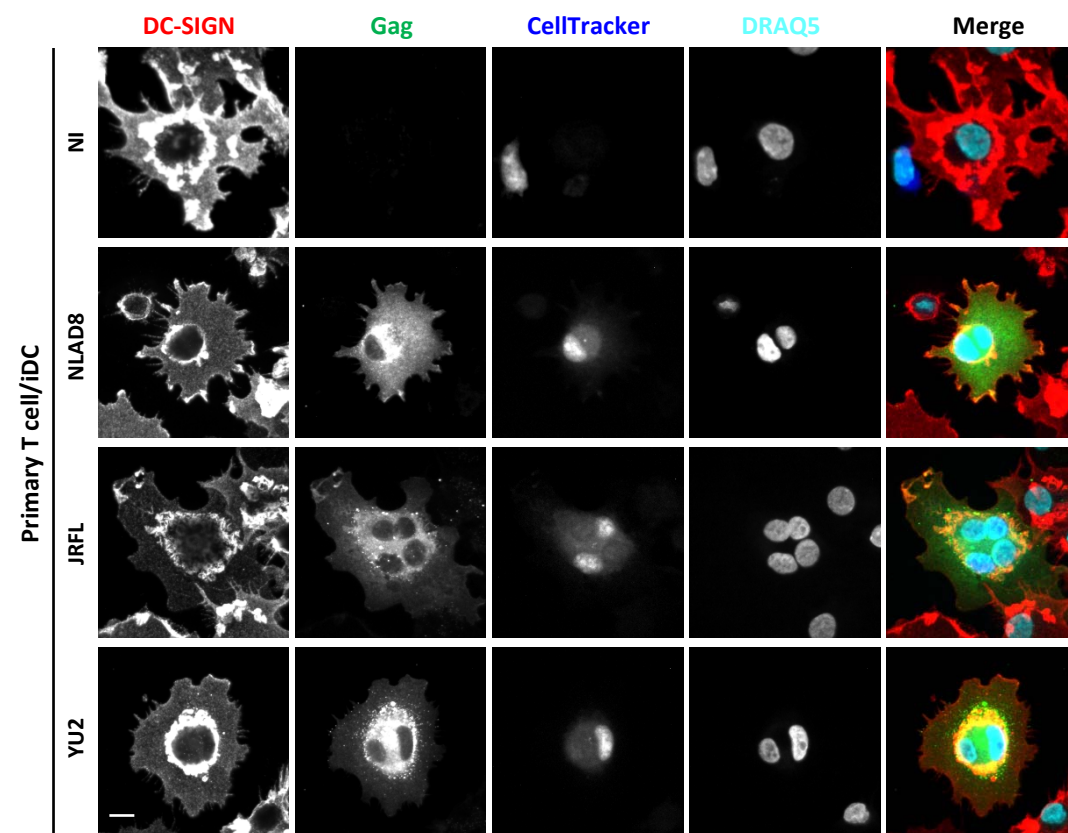**B**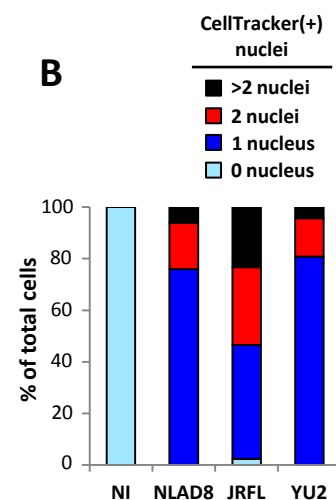**C**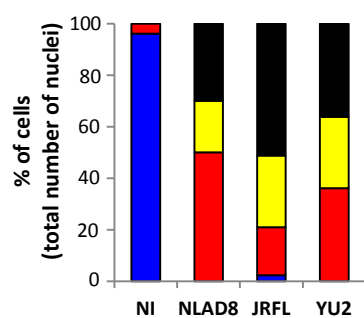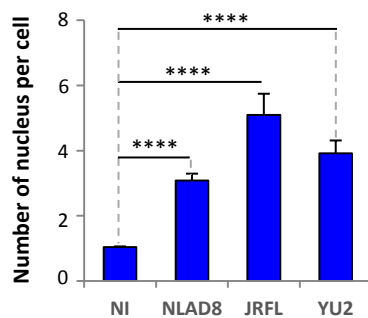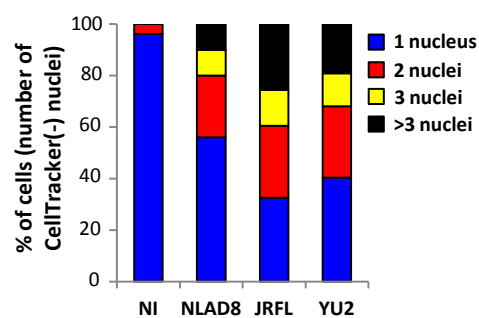

Supplement: FIG S3 [file mBio.02457-19-sf003.pdf]

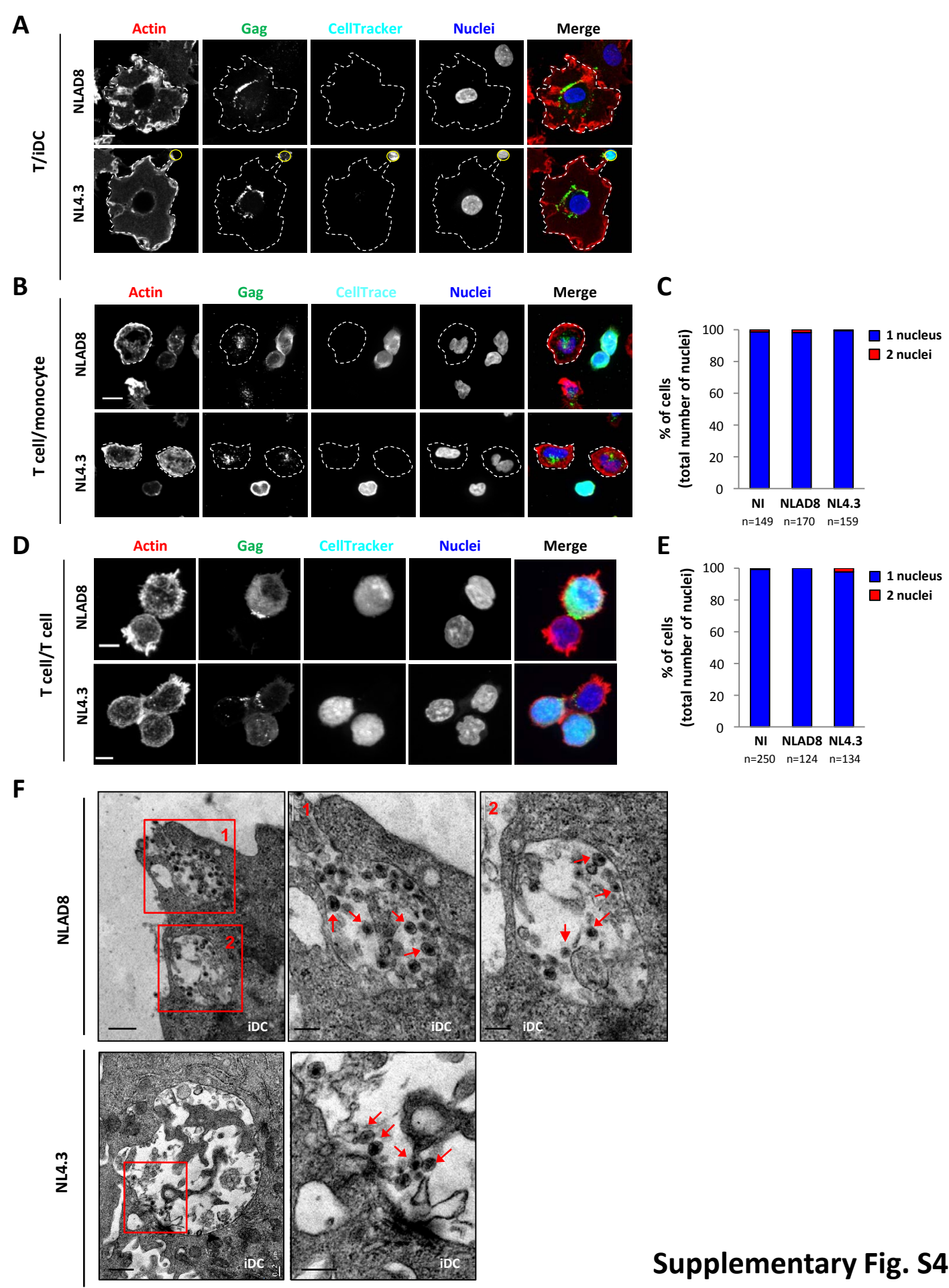

Supplementary Fig. S4

Supplement: FIG S4 [file mBio.02457-19-sf004.pdf]

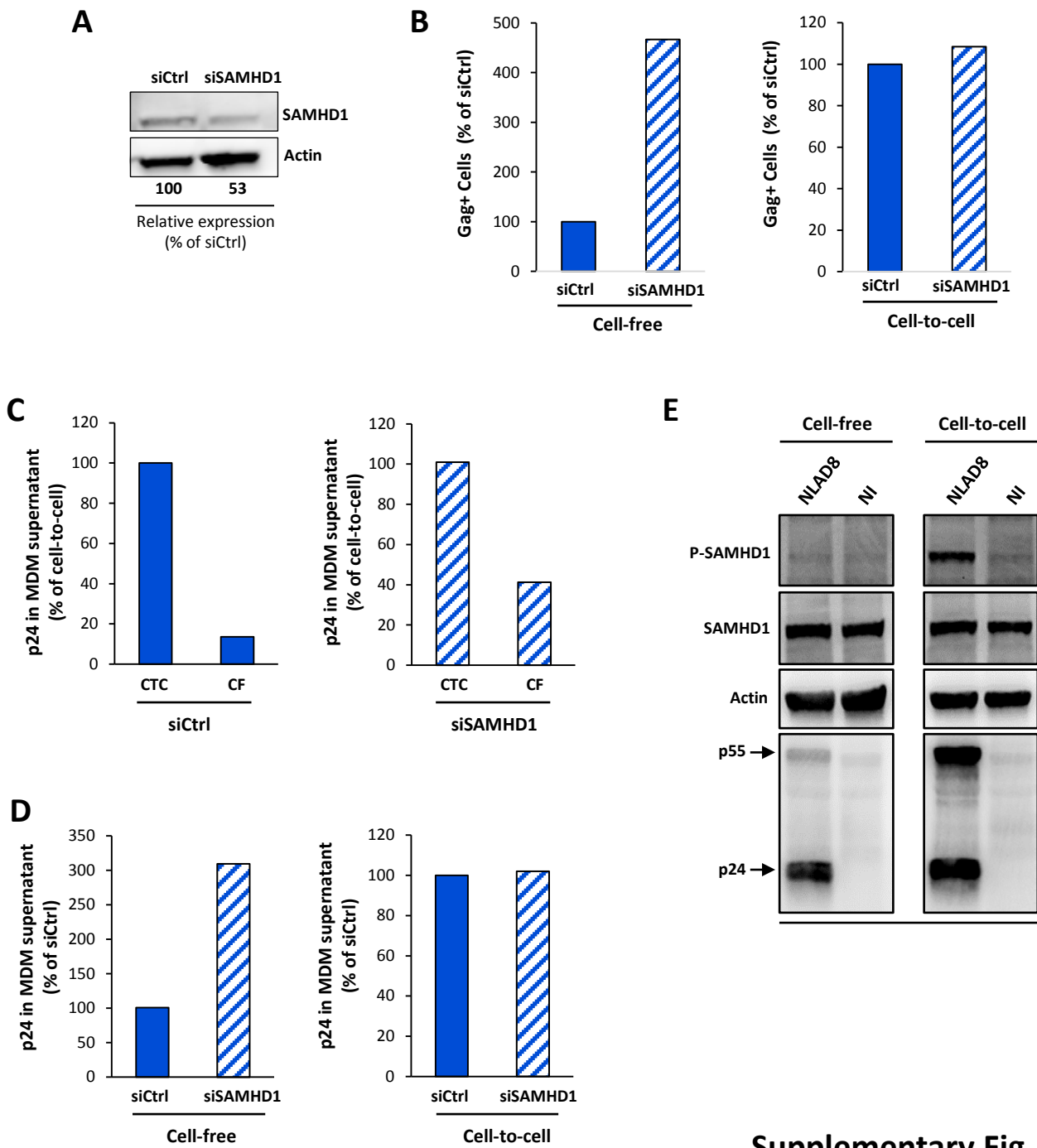

Supplementary Fig. S7

Supplement: FIG S7 [file mBio.02457-19-sf007.pdf]
