# Supplementary material for: Cell-to-Cell Spreading of HIV-1 in Myeloid Target Cells Escapes SAMHD1 Restriction
Source: mBio. 2019 Nov 19;10(6):e02457-19. doi: 10.1128/mBio.02457-19 (PMC6867896; doi:10.1128/mBio.02457-19)
Supplement: FIG S5 [file mBio.02457-19-sf005.pdf]

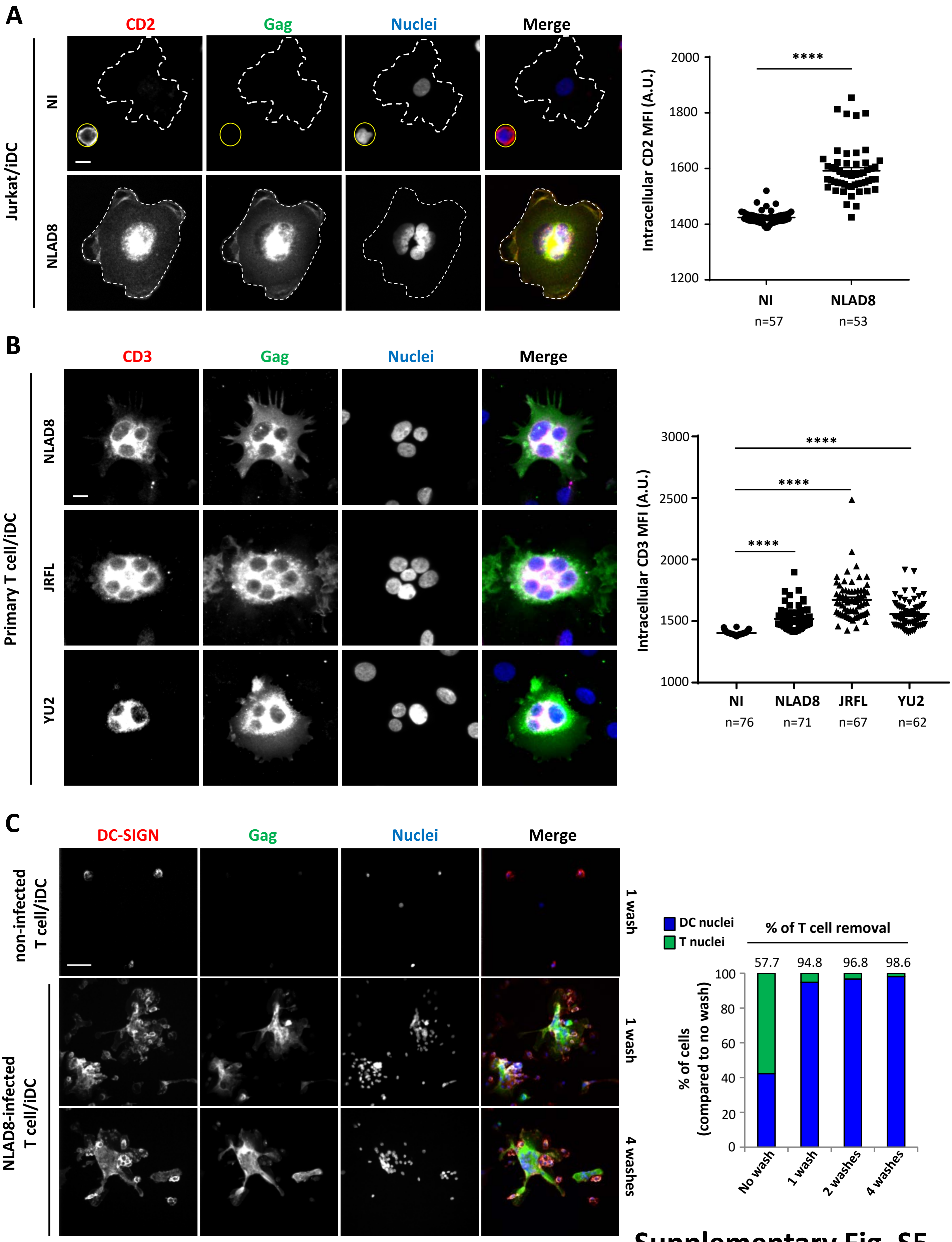

Supplementary Fig. S5
